# Searching the overlap between network modules with specific betweeness (S2B) and its application to cross-disease analysis

**DOI:** 10.1038/s41598-018-29990-7

**Published:** 2018-08-01

**Authors:** Marina L. Garcia-Vaquero, Margarida Gama-Carvalho, Javier De Las Rivas, Francisco R. Pinto

**Affiliations:** 1 University of Lisboa, Faculty of Sciences, BioISI - Biosystems & Integrative Sciences Institute, Campo Grande, C8 bdg, 1749-016 Lisboa Portugal; 2 Cancer Research Center (CiC-IBMCC, CSIC/USAL/IBSAL), Consejo Superior de Investigaciones Científicas (CSIC) and Universidad de Salamanca (USAL), Salamanca, Spain

## Abstract

Discovering disease-associated genes (DG) is strategic for understanding pathological mechanisms. DGs form modules in protein interaction networks and diseases with common phenotypes share more DGs or have more closely interacting DGs. This prompted the development of Specific Betweenness (S2B) to find genes associated with two related diseases. S2B prioritizes genes frequently and specifically present in shortest paths linking two disease modules. Top S2B scores identified genes in the overlap of artificial network modules more than 80% of the times, even with incomplete or noisy knowledge. Applied to Amyotrophic Lateral Sclerosis and Spinal Muscular Atrophy, S2B candidates were enriched in biological processes previously associated with motor neuron degeneration. Some S2B candidates closely interacted in network cliques, suggesting common molecular mechanisms for the two diseases. S2B is a valuable tool for DG prediction, bringing new insights into pathological mechanisms. More generally, S2B can be applied to infer the overlap between other types of network modules, such as functional modules or context-specific subnetworks. An R package implementing S2B is publicly available at https://github.com/frpinto/S2B.

## Introduction

Disruption of a gene sequence may cause the dysfunction of the encoded protein, which can trigger the onset of a disease. Such genes are defined as disease causal genes. Nevertheless, a disease is a pathologic phenotype resulting from synergic disruptions of varied cellular functions caused by both genetic and environmental factors^1^. Consequently, disease associated genes (hereinafter called Disease Genes (DGs)) are not necessarily causal. They can be *modifiers*, that modulate disease severity, or *phenotypical*, unable to influence the disease course but responsible for disease phenotypes. Genes associated with a disease are more prone to interact with each other than with non-disease related genes, establishing network disease modules^2,3^. Disease modules are neighborhoods of the full interactome network containing all disease associated proteins^4^. As interactomic maps are still incomplete^5^ and the number of known DGs is limited^6^, the identification of DGs remains an important issue, contributing to decipher molecular mechanisms of disease and to discover biomarkers and therapeutic options.

Efforts to complete protein interactions networks include not only high troughput experimental approaches^7^, but also computational predictive methods, recently reviewed by Kotlyar *et al*. The latter can be based in sequence features, conservation across species, protein domains, 3D structure, interaction network topology, or a combination of several of the previous data types^8^. To expand the list of known DGs, information systems, like DisGeNet^9^, Open Targets^10^ or DISEASES^11^, integrate and weight heterogeneous evidence sources linking genes with diseases, including text-mining approaches.

Network-based DG prioritization methods aim to recover complete disease modules, using network interactions of known DGs to predict new DG candidates. One such method, DIAMOnD^4^, starts from the set of known DGs and iteratively adds one node to the disease module. The added node is the more statistically enriched in DGs among its direct neighbors. Other DG prioritization algorithms are based on random walks^12,13^ or diffusion algorithms^14^.

Diseases sharing phenotypes exhibit alterations in similar functional pathways, and their disease modules are more likely to overlap^5,15^. Based on this similarity, researchers have identified common functions among the network neighbors of genes associated with Alzheimer’s and Parkinson’s diseases^16^, and looked for common neighbors of proteins associated with autism spectrum disorders^17^.

However, to our knowledge, there is currently no network-based algorithm aiming to directly predict genes simultaneously associated with two diseases. These can provide hypotheses to explain molecular mechanisms of pathophenotypes shared between two diseases. In addition, these candidates can suggest new therapeutic targets, or provide grounds to repurpose current therapies from one disease to the other. With this aim, we propose a network-based approach called S2B (double specific-betweenness). S2B relies on the assumption that interactors more commonly found on shortest paths linking proteins encoded by genes associated to two diseases must appear in the disease modules overlap. To identify and rank these proteins, S2B employs a specific version of betweenness centrality, which measures how many times a node is involved in a shortest path, focusing specifically on shortest paths linking proteins associated with the two diseases.

A similar network approach has been recently proposed to identify the mediator pathways between DGs and genes differentially expressed between healthy and disease samples^18^. Parallel application of this method to related diseases identified common mediator pathways. However, S2B approaches this problem from a different perspective, as it aims to identify individual proteins that are directly involved in the mechanisms of both diseases simultaneously.

We applied S2B to Amyotrophic Lateral Sclerosis (ALS) and Spinal Muscular Atrophy (SMA), two fatal Motor Neuron degenerative Diseases (MND). The most common form of SMA is caused by recessive mutations in the SMN1 gene, encoding the SMN protein. Numerous causal genes have been reported for ALS, involved in multiple functions such as oxidative stress control (SOD1)^19^, vesicle trafficking (ALS2, FIG. 4, OPTN, VABP, CHMP2B) or proteasomal functions (UBQLN2, VCP)^20^. However, RNA metabolism is the function with the largest subset of MND causal genes (TARDBP, FUS, SETX, ATXN2, HNRNPA1, HNRNPA2/B1, ELP3 in ALS, and SMN1 in SMA)^21,22^. While under debate, protein aggregation and RNA metabolism deregulation are the most accepted hypotheses to explain the MND phenotypes. However, it is very intriguing how such critical events could distinctively affect Motor Neuron (MN) physiology.

Although ALS and SMA present distinct clinical features, they show great phenotypic and molecular similarities, implying a common etiology. Indeed, recent work from our group revealed that key MND causal genes SMN, FUS, TDP43 and SETX show tight physical and functional relationship^23^. In the same vein, this paper shows that S2B predicts cross-disease genes (cDGs), providing new insights into the molecular mechanisms of MND.

## Methods

We considered the prediction of cDGs analogous to the problem of finding the overlap between two network modules when information about module composition is incomplete: consider an undirected graph *G* with two overlapping connected subgraphs *A* and *B*. However, we only know subsets *a* and *b* (seeds) that compose *A* and *B*, respectively. With this incomplete information, we cannot define the set of nodes in the overlap between *A* and *B*. We developed a method that knowing the sets of seeds *a* and *b*, predicts which nodes of *G* are more likely part of *A* and *B* simultaneously. This method is based in the computation of the Double Specific Betweenness score (*S2B*) presented in equation (1).

1 $$S2B(k,G,a,b)=\frac{{\sum }_{i}^{i\in a,i\ne j}{\sum }_{j}^{j\in b,j\ne k}sp(k,i,j,G)\cdot t(i,j,G)}{{\sum }_{i}^{i\in a,i\ne j}{\sum }_{j}^{j\in b,j\ne k}t(i,j,G)}$$

Equation (1) computes auxiliary functions *sp(k*,*i*,*j*,*G)* (equation (2)) and *t(i*,*j*,*G)* (equation (3)).

2 $$sp(k,i,j,G)=\{\begin{array}{c}1\,{\rm{if}}\,d(i,j,G)=d(i,k,G)+d(k,j,G)\\ 0\,{\rm{if}}\,d(i,j,G)\ne d(i,k,G)+d(k,j,G)\end{array}$$

3 $$t(i,j,G)=\{\begin{array}{c}1\,{\rm{if}}\,d(i,j,G)\le avgd(G)\\ 0\,{\rm{if}}\,d(i,j,G) > avgd(G)\end{array}$$

In both equations (2) and (3), *d(i*, *j*, *G)* is the length of the shortest path between the *i*^*th*^ and the *j*^*th*^ nodes of *G*. *sp(k*, *i*, *j*, *G)* is an indicator function with value 1 if node *k* is part of a shortest path between nodes *i* and *j*. *t(i*,*j*,*G)* is an indicator function with value 1 if the length of the shortest path between nodes *i* and *j* is equal or lower than the average shortest path length of *G (avgd(G))*. This path length filter is important to avoid the influence of nodes that are loosely related with the other module. Altogether, it means that *S2B(k*,*G*,*a*,*b)* is the fraction of shortest paths linking a node in *a* to a node in *b* that contain node *k*, with length smaller than the average path length of *G*. Before applying equation (1), nodes present in *a* and *b* simultaneously are discarded as these, by definition, belong to the overlap between *A* and *B*. Therefore, shortest paths starting from these nodes diverge from the overlap, increasing the chances of crossing with other shortest paths outside the overlap region.

We observed that only a small number of nodes in the network achieved high *S2B*. If we plot *S2B* against 1*-quantile(S2B)*, we typically observe an L-shaped curve. To define the threshold value that separates high *S2B* from low *S2B* we apply equation (4). This equation finds the *S2B* that minimizes the distance to the origin in the referred L-shaped curve.

4 $$S2{B}_{t}={{\rm{argmin}}}_{S2B(k,G,a,b)}({(\frac{S2B(k,G,a,b)}{{{\rm{\max }}}_{k}(S2B(k,G,a,b))})}^{2}+{(1-quantile(S2B(k,G,a,b)))}^{2})$$

Besides considering only nodes with high *S2B*, we also implemented two specificity scores (equations (5) and (6)).

5 $$S{S}_{1}=P(S2B(k,G,a,b)\ge S2B(k,G,{a}_{R},{b}_{R}))$$

6 $$S{S}_{2}=P(S2B(k,G,a,b)\ge S2B(k,{G}_{R},a,b))$$

*SS*_1_ is the probability that the *S*2*B* of node *k* with seeds *a* and *b* is equal or higher than the same score computed with random seed sets *a*_*R*_ and *b*_*R*_. A high *SS*_*1*_ means that the *S*2*B* is specific for the initial seed sets. *SS*_*2*_ is the probability that the *S2B* of node *k* in graph *G* is equal or higher than the same score computed with a random graph *G*_*R*_, were nodes maintain their degree but edges are randomly shuffled. A high *SS*_*2*_ means that the *S2B* is specific for the connectivity patterns in *G* and is not a consequence of the high centrality of *k*. To compute each specificity score, 200 random seed sets, or randomized networks were employed. Each randomization contributes to the score of all nodes simultaneously. The computation of S2B and specificity scores took around 22 minutes in a 2.8 GHz Intel Core i7 processor and 8 GB of RAM when using a network with 12424 nodes, 90333 edges, 197 ALS and 48 SMA DGs. A description of the use of S2B method to prioritize cDGs is presented in the supplementary text.

### Data availability

The datasets analysed during the current study are available in OMIM [www.omim.org], DisGENET [www.disgenet.org], APID [apid.dep.usal.es] and Huri [interactome.baderlab.org] repositories. All datasets generated during the current study are are included in this published article (and its Supplementary files).

### Code availability

An R package implementing S2B is publicly available at https://github.com/frpinto/S2B.

## Results

### S2B performance with artificial modules

**Figure 1:**
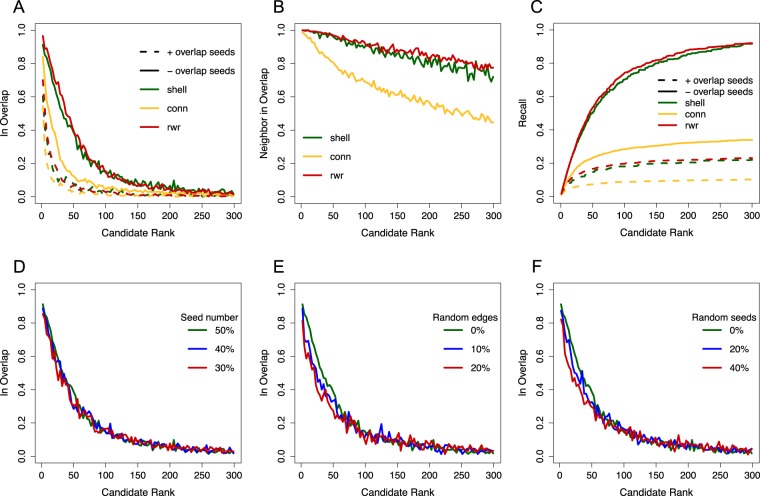
S2B performance with artificial disease modules. (**A**) Fraction of candidates that were in the overlap between modules as a function of *S2B* decreasing rank. **(B)** Fraction of candidates that are direct neighbors of proteins in the overlap **(C)** Recall as a function of *S2B* decreasing rank. Recall is the fraction of proteins in the overlap between the two modules that have an *S2B* rank lower or equal to the candidate rank ploted. In A, B and C three models of disease modules were tested: shell, connectivity (conn) and random walk with restart (rwr) based modules. The impact on method performance of excluding seeds known to be part of both modules was evaluated in A and C. Hereafter, results were computed excluding seeds known to be part of both modules. **(D)** *S2B* robustness upon reduction of the fraction of module proteins used as seeds. **(E)** *S2B* robustness upon randomly rewiring a fraction of network edges. **(F)** *S2B* robustness upon replacing a fraction of input seeds by random proteins. In plots A, B, D, E and F, values are averages of S2B candidates in three consecutive ranks. In A, B and C, 95 pairs of shell modules, 355 pairs of conn modules and 200 pairs of rwr modules were evaluated. In D, E and F, 50 pairs of shell modules were used. Shell modules have between 200 and 400 nodes, while conn and rwr modules have 250 nodes. The overlap between two modules is always between 50 and 125 nodes. In A, B, C, E and F, a 50% random sample of each module was used as seeds.

S2B was applied to random seeds from overlapping artificial modules. Then, the precision and recall in the retrieval of nodes in the overlap region was evaluated. For three different types of artificial modules (see supplementary text), the probability of a node being in the overlap between the two modules decreased for lower *S2B* (Fig. 1A). Figure 1A also confirms that discarding seeds known to be part of the overlap enhances S2B ability to identify top candidates.

The probability of being in the overlap decays rapidly for lower *S2B*. However, as shown in Fig. 1B, candidates maintain a high probability of being direct neighbors of proteins in the overlap for a wider range of *S2B* ranks. *S2B* also correlates with the expected number of direct neighbors in the overlap (Fig. S6A).

Conversely, recall, that is the fraction of all the nodes in the overlap that are correctly predicted in the top ranked S2B candidates, grows almost linearly in the best 50 candidates, and then converges more slowly to its maximum plateau (Fig. 1C).

Figure 1A,B and C show that S2B performs better for random walk with restart (rwr) modules, followed closely by shell modules, both in terms of precision and recall. Performance in connectivity modules is weaker, although maintaining similar trends. S2B performance is similar knowing 50% or only 30% of the proteins involved in disease (Fig. 1D and S6B). We also assessed the impact of false edges in the network (Fig. 1E and S6C) confirming an expected decrease in performance, mainly among the 50 top-ranked candidates. But even when 20% of the network edges are randomly shuffled, prediction quality is not strongly affected. Lastly, Fig. 1F and S6D show that S2B performance is only slightly decreased by inclusion of up to 40% random seeds. Overall, S2B is robust to changes in module topology, incomplete disease characterization, and false positive edges and disease-gene associations.

### Comparing S2B with single disease prioritization methods

To our knowledge, there is currently no other method to predict proteins simultaneously associated with two related diseases (cDGs). However, there are several methods to prioritize genes associated with one disease. We considered applying one of these methods to the seeds of two diseases separately as an S2B alternative. Proteins in the intersection of the two prediction sets would be candidates for simultaneous association with both diseases. We tested this hypothesis with the DIAMOnD algorithm^4^. For each module, 250 iterations were computed and the intersection between the two sets of 250 proteins was compared with the known overlap, estimating DIAMOnD precision (Table 1).

**Table 1:** Precision of DIAMOnD and S2B predictions of proteins in the overlap between pairs of artificial modules.

Module type	# Candidates retrieved by DIAMOND (equal to # top S2B candidates) median [1^st^Q-3^rd^Q]	Precision median [1^st^Q-3^rd^Q]	
		DIAMOnD	S2B
Shell	4 [1–9]	0.00 [0.00–0.18]	1.00 [0.75–1.00]
Connectivity	135 [104–149]	0.60 [0.54–0.73]	0.18 [0.16–0.22]
RWR	8 [1–26]	0.13 [0.00–0.25]	1.00 [0.88–1.00]

Predictions are matched relatively to the number of candidates generated by DIAMOnD for the same pair of modules. 50 module pairs of each type were evaluated.

DIAMOnD predicts many candidates for connectivity modules with moderate precision, while for shell and rwr modules the number of candidates is generally small and precision low. A better performance of DIAMOnD with connectivity modules was expected, as these are generated with the same algorithm used by DIAMOnD to make predictions. For each pair of artificial modules tested, we selected from the top S2B candidates the same number of candidates predicted by DIAMOnD. The matched S2B precisions are higher than DIAMOnD’s for shell and rwr modules, but lower for connectivity modules (Table 1). For this type, the number of DIAMOnD candidates is large and, as shown in Fig. 1A, S2B precision for connectivity modules decays quickly with candidate rank. S2B predictions would have a median precision of 0.60 (similar to DIAMOnD) if the top 20 candidates were considered. In conclusion, although DIAMOnD is a good approach for connectivity type modules, S2B provides a good performance for every type of module tested.

### Identification of common Motor Neuron Disease genes using S2B

To evaluate the potential of S2B, we focused on the Motor Neuron Diseases (MND) Amyotrophic Lateral Sclerosis (ALS) and Spinal Muscular Atrophy (SMA). DGs (seeds) of ALS and SMA (available in supplementary material) were identified from OMIM^24^ and DisGeNET^9^. Human protein interaction networks from two different origins were used. APID (Agile Protein Interaction DataAnalyzer)^25^ gathers literature reported protein interactions, while HuRI (Human Reference Protein Interactome Mapping Project) results from unbiased large scale screens for binary interactions^7,26–29^. Literature-based protein interaction networks are densely connected around proteins of biomedical interest, while large scale experimental techniques may fail to detect interactions between certain types of proteins, such as membrane proteins^30^. In a comparative analysis of S2B results with these networks (supplementary text, Fig S3), it was observed that the fraction of common S2B candidates grows with the level of confidence of protein interactions retrieved from the literature. A mixed APID/HuRI network also shows a high fraction of candidates in common with the separate analysis of the two networks (Fig S3E). Finnally, we opted to merge HuRI with APID interactions reported in a minimum of 3 independent experiments (APID3). This maximizes global interactome and DG coverage while avoids poor quality interactions.

Analysis of 197 ALS and 48 SMA DGs (supplementary data) within the APID3HuRI network returned 232 candidate proteins with a *S2B* higher than *S2B*_*t*_ and both *SS*_1_ and *SS*_2_ higher than 0.90 (supplementary data).

### Comparative Functional Enrichment Analysis of S2B candidates and DGs

S2B candidates should be involved in processes associated with both ALS and SMA DGs (MND-DGs). To assess this hypothesis we performed a comparative Functional Enrichment Analysis (FEA) of Gene Ontology (GO) biological processes associated with S2B candidates and MND-DGs sets. For the latter, only enriched GO terms associated with both ALS and SMA DGs were considered.

MND-DGs and S2B candidates were enriched in 853 and 1110 GO terms respectively. S2B terms contained 43% (392) of the MND-DGs terms. Among the 232 S2B candidates are 5 SMA seeds, 19 ALS seeds and 2 DGs associated with both ALS and SMA (not used as seeds but selected as candidates). Common GO terms could be due to the presence of these seeds among S2B candidates. To evaluate this hypothesis, we performed a randomization test, repeating the FEA with 1000 random sets of 232 proteins extracted from the interaction network, ensuring that 5 SMA DGs, 19 ALS DGs and 2 DGs associated with both ALS and SMA were selected. None of the GO terms enriched in the S2B candidate set was randomly enriched in more than 3.6% of the random sets, showing that S2B GO terms are not significantly biased. Additionaly, the fraction of GO terms enriched in the random sets also associated with MND-DGs was significantly lower than the observed for the S2B candidates (p < 0.001, randomization test).

**Figure 2:**
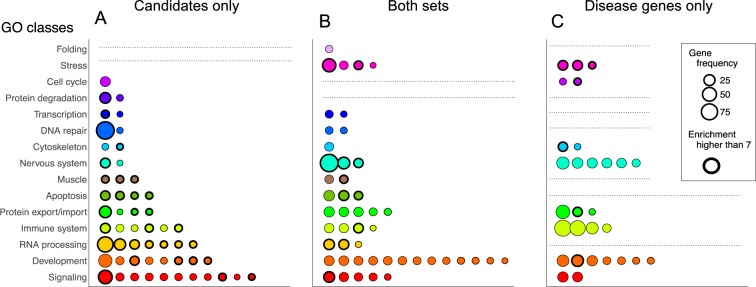
Comparison of functional enrichments between S2B candidates and Disease Genes (MND-DGs) sets. Two independent Functional Enrichment Analyses (FEAs) were performed for S2B candidates and DG sets. FEA results were simplified by merging GO terms into GO groups by gene co-occurrence (if they have 70% of associated genes in common) and semantic similarity (if they have a Lin similarity score higher than 0.70). To further simplify the results, each GO group was assigned to a single GO class by counting the key words most frequent in GO terms descriptions (supplementary text). 67 GO groups were not related to any GO class and therefore were discarded. **(A)** GO groups related only to S2B candidates genes. **(B)** GO groups related both with S2B candidates and with MND-DGs. **(C)** GO groups related only with MND-DGs. Each dot represent a single GO group characterized by the sum of gene frequencies (dot size). GO groups with a 3rd quartile fold enrichment higher that 7 are highlighted with bold border.

Among biological processes uniquely enriched in S2B candidates or in MND-DGs there were still similar processes. Therefore, we applied a simplification workflow (supplementary text) minimizing redundancy by merging them as GO groups (according to overlap between gene sets and to semantic similarity). We further simplified the results by assigning GO groups to functional classes. Finally, we divided GO groups into three sets; GO groups containing only MND-DGs, S2B candidates or both (Fig. 2).

Functional simplification generated 131 GO groups, 48 common to both S2B candidates and MND-DGs sets (Fig. 2B), representing 62% of the MND-DGs GO groups and covering 13 out of the 15 GO classes. Removing term redundancy further increased the recovery of MND-DGs processes by S2B candidates. There are still many GO groups that belong to unique sets (Fig. 2A,C), but most belong to GO classes that are represented in both S2B candidate and MND-DGs sets. The exceptions are two groups of the ‘Protein Degradation’ class, which are only enriched in S2B candidates. Interestingly, protein degradation is a relevant pathway for neurodegeneration and has been previously associated with ALS^31^.

S2B candidate GO groups have higher fold enrichments (ratio between frequency of GO term in the gene list and frequency of the same GO term in the background (the human genome)) than MND-DGs unique GO groups (bold border dots in Fig. 2A,C). Although MND-DGs set gathers the highest number of nervous system-related groups (Fig. 2C), these have lower fold enrichment when compared with those present in both S2B candidates and MND-DGs sets (Fig. 2B). S2B stronger associations are possible due to the higher specificity of processes enriched in the candidate set.

Overall, FEA of S2B candidates identifies biological processes similar to those found simultaneously in ALS and SMA DGs. However, S2B has a higher capacity to uncover specific processes linked to MND phenotypes.

S2B candidates are also significantly enriched in genes associated with neurological, mental and muscular diseases (supplementary text). This association is an independent observation supporting S2B ability to identify genes in disease module overlaps.

### S2B candidates are enriched in DGs simultaneously associated with ALS and SMA identified from different sources

To further validate S2B predictions, we searched for different evidence sources from which DGs for ALS and SMA could be retrieved. We collected DGs from Open Targets^10^ and DISEASES^11^ and filtered out DGs that were in common with DisGeNet or OMIM, or that were not mapped in the APID3HuRI interactome. Open Targets, DISEASES and DisGeNet have text mining approaches and some experimental information sources in common, but resulting disease associations are not extensively overlapping. To complement the list of ALS and SMA DGs not used as input for S2B, we performed a pubmed abstract search for all proteins in the APID3HuRI interactome that were not associated with ALS or SMA through DisGeNet or OMIM.

The intersection of these novel DGs sets and the S2B candidate list is reported in Table 2. S2B candidates are significantly enriched for ALS and SMA DGs obtained from the three sources. Particularly relevant, and in agreement with S2B rationale, is the fact that our candidates have a stronger enrichment for DGs associated simultaneously with both diseases. Overall, we found independent evidences that 99 S2B candidates (out of the 206 not previously associated) are associated with ALS or SMA, 37 of which have evidences for association with both diseases (supplementary data).

**Table 2:** Enrichment of S2B candidates in ALS and SMA DGs from diferent evidence sources.

DGs not present in DisGeNet or OMIM		S2B candidates (206 proteins)	APID3HuRI network (10991 proteins)	Fold Enrichment	p-value
Open Targets	ALS	44	1242	1.89	<10^−5^
	SMA	8	152	2.80	0.005
	Both	6	72	4.45	0.001
DISEASES	ALS	4	77	2.77	0.043
	SMA	3	13	12.31	0.017
	Both	1	1	53.35	<10^−6^
Pubmed abstracts	ALS	72	1482	2.59	<10^−6^
	SMA	48	641	3.99	<10^−6^
	Both	37	413	4.78	<10^−6^

Open Targets and DISEASES platforms were queried for ALS and SMA DGs. For the Pubmed abstracts category, a gene was considered associated with a disease if at least one abstract contained the gene symbol and the disease name (“Amyotrophic Lateral Sclerosis” or “Spinal Muscular Atrophy”). Abstract search was performed with the reutils R package. S2B candidates and interactome network nodes that were DGs identified through DisGeNet or OMIM were excluded from this analysis. Fold enrichment is the ratio between DG frequency in S2B candidates and DG frequency in APID3HuRI network. p-values were computed with an hypergeometric test. S2B candidates that are DGs according to these sources and the pmid of the associated abstracts are available in supplementary data.

### S2B candidate interaction network highlights molecular connections between ALS and SMA

**Figure 3:**
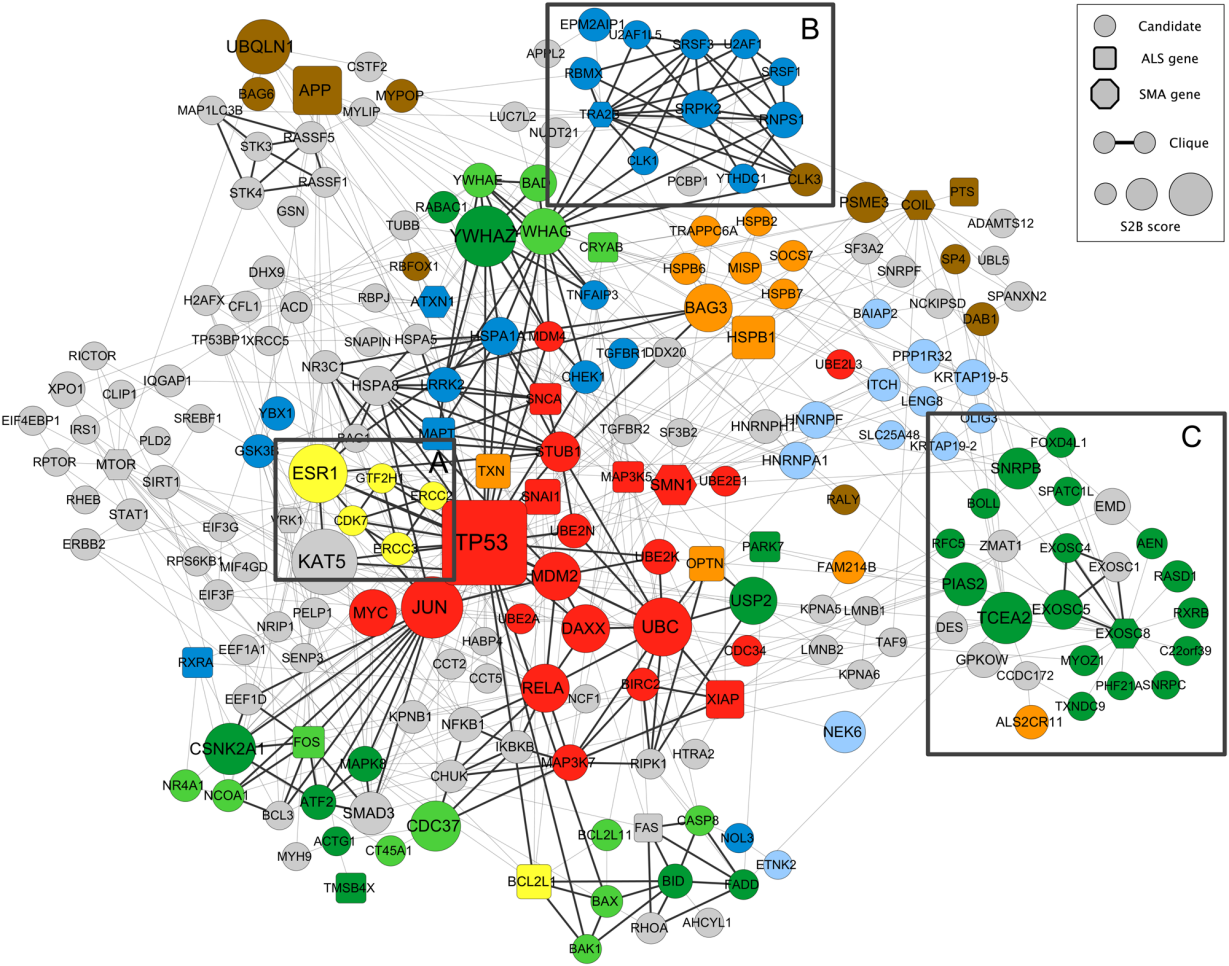
S2B candidate interaction network. Edges represent direct physical interactions between S2B proteins retrieved from the APID3HuRI interactome. Cliques of at least 4 proteins are highlighted with black edges. Clusters formed by proteins that appear frequently together in the shortest paths used by the S2B method (supplementary text) are labeled by node color. **A**, **B** and **C** boxes outline examples in which cliques and clusters overlap. S2B candidates simultaneously identified as ALS or SMA Disease Genes are denoted by node square shape. Node size is proportional to the S2B score.

Seeking mechanistic hypothesis explaining MND phenotypes, we explored the physical interactions between S2B candidates (Fig. 3) recovered from the APID3HuRI interactome. Out of the 232 candidates linking ALS and SMA, 215 are connected in a network component through 603 interactions.

With the S2B candidate subnetwork we aim to demonstrate that our method output is not only a ranked list of proteins. Using the knowledge about the interaction between S2B candidates, we can search for groups of proteins that may be stronger candidates together than individually. We followed two approaches to identify structurally coherent subgroups within S2B candidates. First, we identified cliques (groups in which every protein interacts directly with all other members of the group) with more than 3 elements. The high connectivity of cliques may identify functional complexes. Second, we clustered proteins that co-ocurred in the shortest paths used by S2B (supplementary text). These clusters highlight pathways linking ALS and SMA DGs, suggesting common MND triggering factors.

The first approach returned 75 cliques divided in three connected components (black edges in Fig. 3). The overlap between most cliques demonstrates the high density of interactions among candidates. The second approach returned 8 clusters (labeled by node colors in Fig. 3) with an average size of 17 proteins (ranging from 6 to 33). Interestingly, identified cliques and clusters display frequent overlap, which would be expected if S2B candidates link ALS and SMA disease modules through discrete molecular pathways.

The most coherent overlap is found around the yellow cluster (Fig. 3A), which captures four of the ten subunits of transcription factor TFIIH complex, involved in RNA polymerase II (Pol II) dependent transcription and the DNA Nucleotide Excision Repair (NER) pathway. The TFIIH core complex is formed by 7 subunits, including the ERCC2 and ERCC3 DNA helicases, which help to create the transcription bubble^32^. The activity of RNA polymerase II (Pol II) is induced by anchoring the CDK-activating kinase complex (CAK) to the TFIIH core complex. The CAK subcomplex is composed of MAT1, cyclin H and CDK7. The cluster further contains the GFH2H1 gene encoding the TFIIH-core complex p62 subunit, primarily involved in NER pathway^33^.

A relation between neurodegeneration and DNA damage has been proposed^34^. This connection assumed particular relevance for MND with the discovery of mutations causing a juvenile form of ALS (ALS4) and autosomal dominant proximal spinal muscular atrophy (AOA2) in the gene encoding senataxin (SETX)^35,36^. Senataxin is a DNA-RNA helicase involved in RNA metabolism and DNA integrity maintenance^37^. Strinkingly, Senataxin and SMN protein have been found to collaborate in resolving DNA/RNA hybrids (R-loops), a process that requires tight balance to keep a commitment between correct RNA transcription and DNA damage control^38^. Recently, a growing number of reports point to R-loops and DNA damage as a key commonality between ALS and SMA^39–43^. It is thus quite striking that proteins central to the transcription coupled repair and NER pathways have been selected as top candidates by S2B.

A second cluster highlighted in Fig. 3B also displays a large overlap with a clique group. This group is dominated by splicing-related proteins such as SR proteins (SRSF1, SRSF3), SR-regulating kinases (SRPK2, CLK1, CLK3), general splicing factors (U2AF1, U2AF1L5) and splicing auxiliary components (YTHDC1, RNPS1). The group further includes RBMX and TRA2B (SFRS10), two RNA splicing regulators.

Splicing is one of the critical functions that has been proposed to be altered in SMA, since the best known role for the SMN protein is the biogenesis of the splicing machinery. The SMN protein is further involved in generating the core machinery for other RNA-metabolism related functions including histone mRNA processing and cytoplasmic mRNA turnover^44^.

The connection to splicing was also observed in ALS, as two of the most well studied disease causing mutations involve the TDP-43 and Fus proteins, which both act as splicing regulators^23^.

Splicing regulation relies heavily on multifunctional proteins that tend to establish self-regulatory interaction to control their expression levels. RBMX (also called hnRNPG) and TRA2B are able to act as either activators or repressors of splicing^45^. Interestingly, RBMX has been shown to act together with TRA2B to regulate splicing of the main SMA modifier gene, SMN2^46^.

RNA binding proteins have also been shown to be closely involved in the maintenance of genome integrity and in the response to DNA damage^47^. This seems to involve both the establishment of direct interactions with nascent transcripts to prevent genomic instability, and the regulation of splicing of DNA repair, cell cycle and apoptosis genes. Within the members of this cluster; SRSF1, SRSF3, SRPK2, CLK1, U2AF1, RNPS1, RBMX and TRA2B have all been implicated in this process^47^. These candidates may thus highlight novel elements that disturb RNA processing networks critical for in MND phenotypes.

A third cluster-clique overlap is centered around the RNA exosome complex components EXOSC4, EXOSC5 and EXOSC8 (Fig. 3C). The RNA exosome is a conserved multi-protein complex located in the nucleus and the cytoplasm and is critical for both processing and degradation of various RNAs^48^. Several tissue-specific diseases and complex disorders have been linked to mutations in exosome complex proteins^49^. In fact, EXOSC8 is an SMA associated gene^50^. Interestingly, this clique is integrated in a cluster that captures the SNRPB, SNRPC, PHF21A, and TCEA2 genes, among others.

The SNRPB gene encodes the Sm B/B’ protein, a component of the spliceosomal U1, U2, U3 and U5 small nuclear ribonucleoproteins (snRNPs), the building blocks of the spliceosome. Sm proteins are recognized by the SMN complex, which assembles them in a ring-like structure around the snRNAs, a function that is compromised in SMA leading to changes in the relative proportions of snRNP complexes^33^. The interaction between EXOSC8 and SNRPB (Fig. 3C) goes in line with previous studies reporting that the Sm complex is required for the processing of small non-coding RNAs by the exosome^51^. In contrast to SNRPB, SNRPC encodes a U1snRNP-specific accessory protein. U1snRNP complex interactions have recently been highlighted as an important link between ALS and SMA^23^.

PHF21A (BHC80) also interacts with EXOSC8 (Fig. 3C). It is a component of histone deacetylase BHC complex and mediates transcriptional repression of neuron-specific genes in non-neuronal cells^52^. Conversely, PHF21A protein recognizes H3K4 specific methylation states, an histone that is associated to neurodevelopmental diseases such as Autism Spectrum Disorders^53^. It is known that histone biogenesis disturbance may contribute to the etiology of SMA since low levels of SMN affect U7snRNP biogenesis and, in consequence, histone mRNA processing^54^. This cluster reveals that MND phenotypes might be also influenced by tissue-specific chromatin deregulation events.

The cluster surrounding EXOSC8 further includes the transcription elongation factor TFIIS encoded by TCEA2. TFIIS is a critical factor for efficient transcription elongation and interestingly, a top 10 ranked S2B candidate (Fig. 3C). TFIIS directly binds Pol II to stimulate its release from promoter proximal positions and thereby produce full length transcripts^55^.

Thus, this cluster reveals strong links between RNA transcription, processing and turnover. On the other hand, recent results highlight important functions for the nuclear exosome in the response to DNA damage, including direct interactions with the Senataxin protein, which acts as an exosome co-factor for sites of transcription-induced DNA damage^56^.

The examples used for detailed exploration of the S2B candidate network (Fig. 3A–C) were selected based solely on structural reasons. However, they outlined a tight relationship between RNA homeostasis (transcription, splicing and degradation) and DNA damage repair that, together with the previous knowledge about ALS and SMA DGs, supports its implications on MND etiology. We believe this analysis demonstrates S2B usefulness to predict protein candidates linking ALS and SMA and furthermore, suggest potential mechanisms that explain the molecular relation between the two diseases.

## Discussion

S2B is built upon the hypothesis that disease genes tend to interact in cellular networks within disease modules and that related diseases have an overlap between their modules. The frequency with wich nodes belong to shortest paths between nodes associated with two related diseases (cDGs) allows the detection of specific nodes bridging disease modules.

S2B performance with artificial modules shows that nodes with high *S2B* have a high likelihood of belonging to the overlap between modules. Moreover, this predictive capacity is robust to changes in module topology, both to the quantity and the quality of the input DGs and network interactions. Our results with artificial modules also support the use of S2B to predict the overlap between network modules of varied type, such as functional modules or context-specific subnetworks.

In the artificial module analysis, we generated and controlled the complete composition of each module, and selected for analysis pairs of modules with overlap. In this selection, we did not control for the presence of network hubs in the overlap. For this reason, applying the specificity thresholds in the analysis of artificial modules should not bias the method performance. Concordantly, it can be observed in Fig. S5(A and C) that proteins with higher S2B values are not biased to pass the filters for both specificity scores.

Network hubs can indeed be part of the overlap between real disease modules and have a significant role connecting the mechanisms of both diseases. However, they are not interesting candidates for follow up studies, since they tend to be unspecific and simultaneously related with many different cellular processes. Therefore, specificity score filtering is important for the analysis of real disease seed sets.

In the study of ALS and SMA, S2B successfully returned candidates involved in processes known to be part of motor neuron degeneration mechanisms, such as apoptosis, DNA repair, RNA processing, protein transport or cytoskeleton organization^23^. More specifically, S2B candidates were enriched for DGs simultaneously associated with ALS and SMA through different information sources and not used as input for S2B predictions.

Some of the cliques and clusters in the candidate interaction network were involved in several of these processes, which suggests that disease proteins tend to be located at the interface between functional modules and corroborates that disease modules do not overlap perfectly with functional and topological network modules^4,57^.

Many of the S2B candidates were already associated with multiple diseases, some of them closely related with ALS and SMA. Together with the observation that most candidates interact in a densely connected network, these results reinforce the hypothesis that DGs tend to interact with other DGs, specially if the two diseases are related through similar causes or phenotypes^15^.

S2B can be applied to uncover common molecular mechanisms shared by various diseases. Its discovery potential can be amplified through the use of different networks types, such as signaling and gene regulatory networks, and by integrating genome scale molecular data characterizing healthy and disease states.

In summary, this work provides a novel approach to predict the overlaps between network modules, which can uncover disease mechanisms through network exploration for pathologies with phenotypic similarity. Its application to the motor neuron diseases SMA and ALS identified several novel genes as potentially involved in critical pathomechanisms, opening new hypothesis for experimental exploration.

## Electronic supplementary material

Supplementary text

Supplementary Data
