# Supplementary material for: Searching the overlap between network modules with specific betweeness (S2B) and its application to cross-disease analysis
Source: Sci Rep. 2018 Aug 1;8:11555. doi: 10.1038/s41598-018-29990-7 (PMC6070533; doi:10.1038/s41598-018-29990-7)
Supplement: Supplementary file 1 — Supplementary text [file 41598_2018_29990_MOESM1_ESM.pdf]

Supplementary Methods and Results

Searching the overlap between network modules with specific betweenness (S2B) and its application to cross-disease analysis.

Marina L. Garcia-Vaquero[1], Margarida Gama-Carvalho[1], Javier De Las Rivas[2], Francisco R. Pinto[1*]

[1] University of Lisbon, Faculty of Sciences, BioISI – Biosystems & Integrative Sciences Institute, Campo Grande, Lisboa, Portugal

[2] Cancer Research Center (CiC-IBMCC, CSIC/USAL/IBSAL), Consejo Superior de Investigaciones Científicas (CSIC) and Universidad de Salamanca (USAL), Salamanca, Spain

Supplementary Methods

Retrieval of Disease Genes

We retrieved all the ALS and SMA disease genes (DGs) described on OMIM (Online Mendelian Inheritance in Man; (https://www.nlm.nih.gov/mesh/MBrowser.html)[1] and DisGeNET; (http://www.disgenet.org)[2] databases in September 2016 (supplementary data). In both cases, we took all the available associations without any quantitative filtering. We also merged associations for available subtypes of each disease.

PPI data collection and network construction

Human physical Protein-Protein Interaction (PPI) data was extracted from HuRI (Human Reference Protein Interactome Mapping Project) (interactome.baderlab.org)[3–7] and APID (Agile Protein Interaction DataAnalyzer) (apid.dep.usal.es/)[8] databases (accessed in February 2017). We constructed undirected and unweighted networks using Igraph R-package[9]. Loop and multiple edges were eliminated and only the main component of the network was selected. Finally, ALS and SMA DGs were labeled as seed nodes.

Artificial disease modules

Three different types of modules were used, based on distinct hypothesis for the spread of disease-causing perturbations across cellular networks. Shell modules[10] are composed by a seed node and all other nodes in the network at distance of 2 or lower. These artificial modules assume that the perturbation spreads homogeneously through the network. Connectivity modules[10] are built iteratively around a seed node, adding at each step the node most significantly enriched in links to previous module members. These modules assume that disease perturbations affect predominantly nodes that are specifically linked to causal genes. Random walk with restart (rwr)[11] modules simulate the path of an imaginary walker that, at each time step, moves to a randomly chosen direct neighbor or, with a given restart probability, returns to the seed node. The nodes with higher probability of being visited by the walker constitute the model. These modules assume that disease perturbations spread more easily to nodes with multiple and shorter paths linking to the causal nodes. Real disease modules can be a mixture of these and other module types, as the disease perturbation pattern along the network may depend on the type of molecular function of each protein and the nature of each protein-protein interaction.

Artificial disease modules were constructed using the APID3 protein interaction network. Each seed originated three artificial modules with different topological properties. Shell modules were composed by the seed and proteins at distance 1 or 2 in the network. Only shell modules with more than 200 and less than 400 proteins were kept. To increase the probability that shell modules reached this size range, only proteins with a degree between 19 and 22 were selected as possible causal seeds. Connectivity modules were composed by the seed and 249 proteins added iteratively. In each step, all proteins out of the growing module were tested for having a higher than expected number of links to proteins in the module using an hypergeometric test. The protein with the smallest p-value was added to the module. Random walk with restart modules were composed by the 250 proteins with higher occupancy probability in the random walk stationary distribution initiated in the seed node with a restart probability of 0.75. The stationary distribution was determined numerically as previously described[11]. Within each topology type, existence of overlap between all possible module pairs was evaluated. Only module pairs where the overlap contained between 50 and 125 proteins were used to test S2B performance.

Functional enrichment comparison

We performed a comparison of Functional Enrichment Analyses (FEAs) of MND-Disease genes (MND-DGs) set and S2B candidate genes. The initial gene sets entailed 370 MND-DGs (295 ALS and 93 SMA genes, being 18 common to both diseases) and 232 S2B candidate genes. Functional enrichment of Disease Genes gathers only the GO terms that were associated to at least one ALS and SMA gene simultaneously. Both sets were functionally enriched for Gene Ontology Terms (GO) Biological Process (BP) using EnrichGO R-package[12]. Results were filtered using an adjusted p-value ≤ 0.05 and human genome background frequency ≤ 0.10. To better reflect the statistical significance of the results; we calculated the fold enrichment of each GO term (ratio between the frequency of the GO in the gene list and the frequency of the same GO in the human genome background gene list). The FEAs returned 461 and 718 enriched GO terms for MND-DGs and candidates respectively. Due to the Gene Ontology (GO) hierarchical structure, when a GO term is enriched it is likely that some of its ancestors are also enriched, increasing the results size and redundancy. In order to facilitate the analysis of the results, we applied a simplification workflow (Fig S1B). We firstly created GO groups of GO terms showing gene co-occurrence (at least an overlap of 70% of associated genes) and semantic similarity (GO terms that presented a Lin's semantic similarity score ≥ 0.70) (Fig S1B-1). This approach finds "hidden" commonalities between apparently different GO terms, thus it

was applied jointly for both FEAs. When a GO group was formed, it retained the 3rd quartile of fold enrichment and the sum of gene frequencies of the merged GO terms for candidate and Disease gene sets respectively (Fig S1B-1).

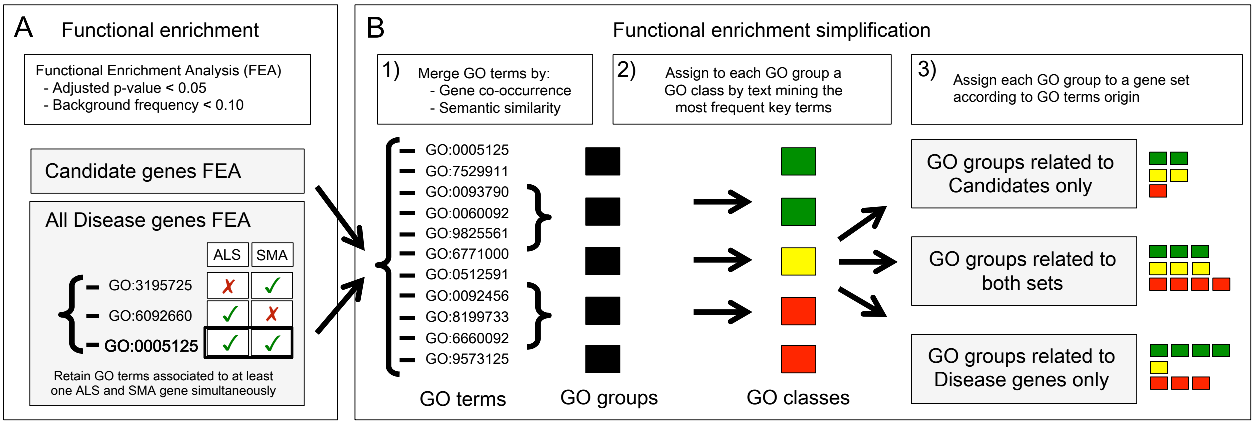

Fig S1. Functional enrichment analysis (FEA) simplification and comparison workflow. A) FEA of S2B candidate and Disease Genes (MND-DGs) sets. MND-DGs FEA gathers only the GO terms that were associated to at least one ALS and SMA gene simultaneously. B) Functional simplification; 1) Resulting GO terms are merged into GO groups by gene co-occurrence and semantic similarity, 2) GO groups are classified based on the most recurrent key term and 3) GO groups are assigned to a final set according to the genes associated to each GO term in the respective GO group.

Table S1 – GO classes and corresponding key terms used to define them through text mining of GO terms.

	GO class name	Key terms
**1**	Nervous system	neuron, synaptic, axon, microglial, glial, neural, neuromuscular, neurogenesis, nervous
**2**	Immune system	immune, host, pathogen, interferon-beta, cytokine, fungus, interleukin-2, interleukin-1, leukocyte
**3**	Muscle	Muscle
**4**	Stress	stress, heat, oxidative, UV, X-ray, superoxide
**5**	Folding	aggregation, folding
**6**	Apoptosis	apoptosis, apoptotic, autophagy
**7**	Cytoskeleton	cytoskeleton, microtubule, actin
**8**	RNA processing	RNA, processing, mRNA, spliceosomal, splice
**9**	Transcription	transcription, chromatin, histone
**10**	DNA repair	DNA, repair
**11**	Protein degradation	degradation, proteolysis, ubiquitination, deubiquitination, ERAD
**12**	Cell cycle	cycle, mitotic, cytokinesis
**13**	Protein export/import	localization, transport, import, export, targeting
**14**	Signaling	transduction, cascade, signaling, signal
**15**	Development	development, developmental, differentiation, embryo, embryonic, morphogenesis

Due to the heterogeneity of biological processes retrieved, we manually created 15 major functional classes (GO classes) defined each one by a set of key words described in Table S1. Then, each GO group was assigned to the GO class most represented in the contained GO terms' descriptions (Fig S1B-2). Finally, GO groups were divided in three sets according to; if they had GO terms associated only to candidate genes, to MND-DGs or to both initial gene sets (Fig S1B-3).

Analysis of shortest path clusters in S2B candidate interaction network

The physical interactions between S2B candidates were retrieved form the APID3HuRI interactome, generating an S2B candidate interaction network. We generated clusters of candidates that tend to be part of the same shortest paths linking seed proteins. First, we gathered the list of shortest paths used in S2B computation and containing each candidate. For each pair of candidate proteins, we computed a jaccard coefficient evaluating the ratio of the number of shortest paths where both candidates were present together over the number of shortest paths where at least one of the candidates was present. Pairs of candidates with a jaccard coefficient greater than 0.25 were linked in a network. The clusters were expanded to include all the candidates that were present in 75% or more of the shortest paths containing the initial cluster members. Connected components with more than 3 candidates or isolated cliques with 3 members were selected to generate a candidate cluster.

Supplementary Results

Double specific-betweenness (S2B)

Double specific-betweenness (S2B) is a network analysis method tailored to take advantage of diseases known to have common phenotypes and predict novel cross-disease associated genes (cDGs). The principle behind S2B is that network paths connecting a protein associated with one disease to a protein associated with the other disease should go through proteins in the overlap between disease modules. Therefore, if we analyze all the known shortest paths linking one disease module to the other, the more frequent members of those shortest paths are very likely in the overlap between disease modules.

The S2B method main inputs are protein interaction networks and lists of Disease Genes (DGs) known to be associated with the two diseases (seeds) (Fig S2A). The core of the method is the computation of a version of Betweenness centrality measure - number of

times a protein is part of a shortest path - that is specific for the lists of DGs (Fig S2B). For each node in the network, S2B counts the number of times the node is part of a shortest path between proteins encoded by Disease A Genes to Disease B Genes. Shortest paths longer than the networks average path length are not included to avoid the influence of proteins loosely related to one of the diseases (yellow nodes in Fig S2B).

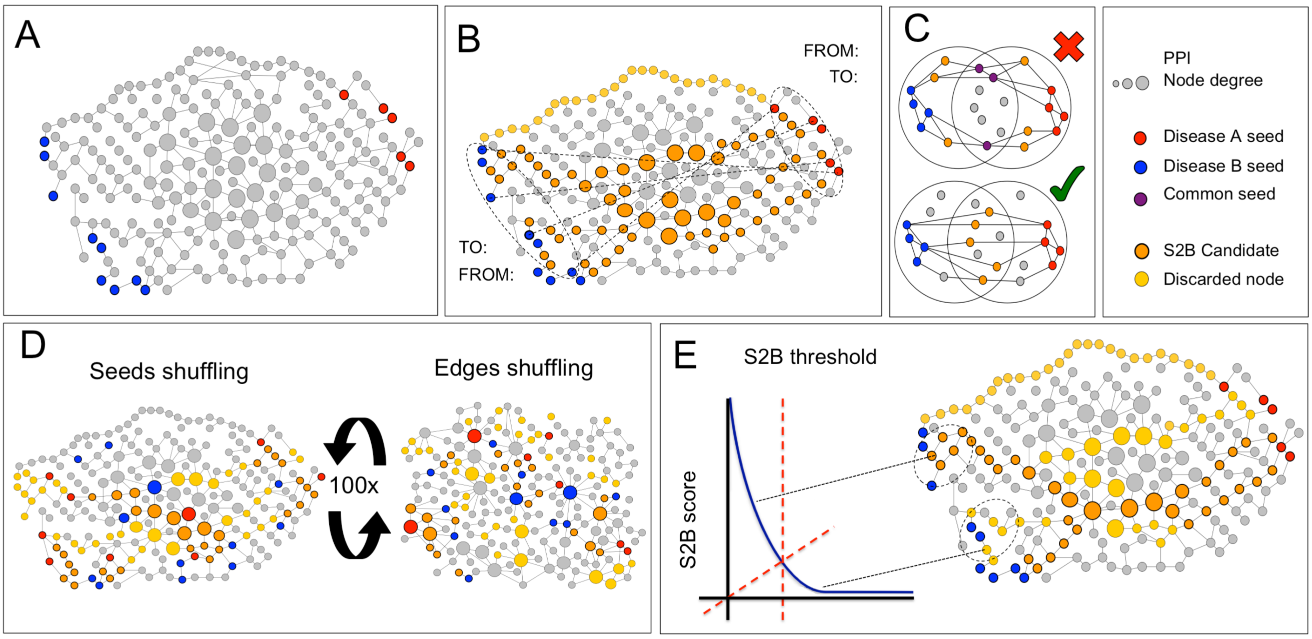

Fig S2. Overview of the S2B method: (A) Human interactome network construction. A global interactome network is constructed using physical protein interaction (PPI) data. Seeds of Disease A and B are identified using gene-disease association data. (**B)** Specific Betweenness Count. The S2B method exploits a novel version of Betweenness centrality measure that counts the number of times a node is involved in a shortest path linking Disease A to Disease B seeds. Shortest paths longer than the network average path length are excluded to avoid the influence of loosely related proteins (yellow nodes). (**C)** Seed filtering. In order to improve S2B performance, genes associated simultaneously to both diseases are discarded. (**D)** Specificity scores (SS). The S2B method includes two specificity scores derived from two types of randomizations that measure how many times a node has a higher specific *S2B* in the original interactome than in randomized networks. The first randomization consist on shuffling the identity of seeds while preserving network structure (1D). In the second, all network edges are shuffled maintaining the degree of nodes in the network (2D). (**E)** S2B candidates selection. First, *S2B* is normalized by dividing it by the number of shortest paths, shorter than the average path length, linking seeds in the network. Then, the *S2B* threshold is defined as the point at which ranked *S2B* decrease rate shifts upwards (described in methods). Final S2B candidates are those proteins that have both SS higher than 0.90 and overcome the *S2B* threshold.

Proteins associated to both diseases are also discarded as these proteins, by definition, belong to the disease modules overlap (Fig S2C). Therefore, shortest paths starting from these proteins diverge from the overlap, increasing the chances of crossing with other shortest paths outside the overlap region.

A second layer of specificity is introduced by evaluating if some nodes have high specific *S2B* just because they are very central in the network. To detect these nodes, *S2B* is recomputed in randomized networks (Fig S2D). If random *S2B* values have similar or higher values than the original *S2B*, then the node is not specifically linking the two sets of seeds. Although these nonspecific nodes can be part of the disease module overlap, they would probably have high *S2B* for many different diseases or if the seeds were random sets of proteins. Specificity Scores (SS) are measured as the fraction of randomized networks yielding lower *S2B* when compared with the original network. Two sorts of network randomization are employed, either by randomly permuting seed protein identity or by shuffling network edges maintaining node degree (number of incident edges). The first method allow us to ask if nodes with high *S2B* are specific for the seeds used, while the second method asks if high *S2B* values are specific for particular pathways in the network.

To enhance the comparability of *S2B* values across different networks or input seed sets, we compute a normalized *S2B* that results from dividing *S2B* values by the total number of shortest paths between seed nodes smaller than the average path length. During the method development we observed that the distribution pattern of *S2B* across the nodes in the network is invariant. If *S2B* are plotted in decreasing order, an L-shape is observed (Fig S2E). This means that there is a small fraction of nodes with high *S2B* while most of the network nodes have very small scores. We define an *S2B* threshold that divides the L-shaped curve in two parts, finding the point that is closest to the origin of the plot (described in methods). To the left of that point we find the set of nodes in the network that accumulate the highest *S2B*. S2B candidates are required to have both SS higher than 0.90 and a *S2B* higher than the *S2B* threshold (orange nodes in Fig S2E).

Identification of common Motor Neuron Disease genes using S2B

To evaluate the potential of application of the S2B method, we decided to focus on the Motor Neuron Diseases (MND) Amyotrophic Lateral Sclerosis (ALS) and Spinal Muscular Atrophy (SMA) as a case-study. There are numerous ALS and SMA Disease Genes (DG), known to be involved in closely related functions. The genotypic and phenotypic similarities between MND suggest that the ALS and SMA disease modules overlap. The S2B method could therefore help to further define the MND molecular landscape and possibly identify key elements responsible for triggering MN degeneration.

The first step of the S2B method is to map known MND-DGs (seeds) onto interaction networks (supplementary data). Considering that different networks are currently available for the human interactome, we first began by assessing how S2B predictions can be influenced by the source type and quality of the interaction data used. Thus, the S2B method was applied to human protein interaction networks from two different origins. The APID (Agile Protein Interaction DataAnalyzer) repository[8] gathers protein interactions reported in the literature, while the HuRI (Human Reference Protein Interactome Mapping Project) database is the result of unbiased large scale screens for binary interactions between human proteins[3–7]. Literature-based protein interaction networks are more densely connected around proteins of biomedical interest, while large scale experimental techniques may fail to detect interactions between certain types of proteins, such as membrane proteins[13]. Both kinds of biases may condition S2B candidate selection. In the case of APID interaction data, three networks with increasing degree of confidence were assembled by only including interactions described in a minimum of two (APID2), three (APID3) or four (APID4) independent experiments. We compared the fraction of common seeds and S2B candidates among the four networks, taking into account the different network properties and intersections (Fig S3).

APID networks of higher confidence are completely contained in APID networks of less quality (Fig S3A and S3B). The intersection between APID networks and HuRI is high in terms of proteins but low when edges are considered, which reflects the dissimilarities between different approaches for PPI detection. The presence of a higher number of MND-DGs in APID versus the HuRI network (Fig S3C) is in agreement with the underlying network generation procedures – literature based for APID *versus* unbiased screening for HuRI.

As expected, the increase on S2B Specificity threshold always induces a decrease on the fraction of S2B candidates common to the compared networks (Fig S3D). S2B candidates may differ between networks because the input lists of MND-DGs vary between networks. Likewise, different nodes and edges may reroute shortest paths between disease proteins. Even if for some nodes the shortest paths are conserved across networks, changes in one network context may lower specificity scores and change candidate selection.

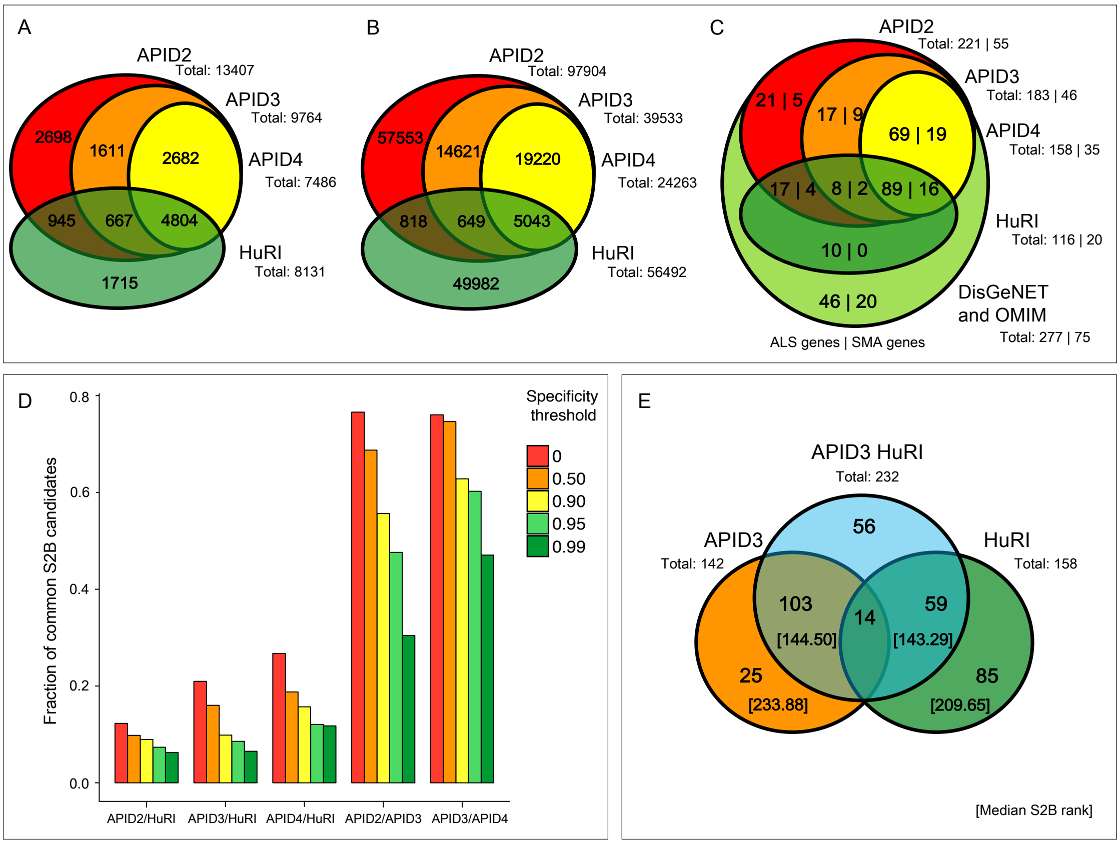

Fig S3. Impact of network characteristics on S2B method predictions. Four protein interaction networks were constructed using different source and quality data. HuRI is constructed with unbiased high throughput-derived binary interactions whereas APID networks combine literature-derived and experimentally validated data. APID2, APID3 and APID4 include interactions described in a minimum of two, three and four independent experiments, respectively. **(A)** Intersection of nodes between networks. **(B)** Intersection of edges between networks. **(C)** Intersection of mapped ALS and SMA DGs between networks. DGs associated to ALS and SMA simultaneously were discarded. **(D)** Fraction of S2B candidates in common among input interaction networks and according to the specificity threshold used. The fraction of common S2B candidates among networks was computed using the smallest network candidate list as a reference. **(E)** Intersection between S2B candidates obtained using APID3, HuRI or the merged network APID3HuRI. The median rank of the S2B candidates found only in APID3 or HuRI were compared against those simultaneously selected using the APID3HuRI network.

The APID2/APID3 and APID3/APID4 network pairs display the highest number of common candidate genes in the absence of a specificity threshold (Fig S3D). Interestingly, the APID3/APID4 overlap is more robust to increasing specificity thresholds, likely reflecting the greater interaction quality of the underlying networks. Likewise, despite of the intrinsic dissimilarities between the HuRI and APID networks, the fraction of HuRI S2B candidates in common with APID increases with interaction quality for all specificity thresholds (Fig

S3D). These results suggest that the removal of less reliable interactions has a positive impact on the S2B method capacity to identify the best candidates. On the other hand, higher quality networks are smaller, leading to lower number of mapped DGs (Fig S3C) and the consequent loss of input information for the method.

To maximize global interactome and DG coverage while avoiding poor quality interactions, we opted to merge HuRI and APID3 networks for subsequent analysis. Moreover, the sizes of APID3 and HuRI networks are more similar, which allows a balanced mix of data derived from high-throughput experiments and literature knowledge.

The S2B method applied to 197 ALS and 48 SMA DGs within the APID3HuRI network returned 232 candidate proteins potentially related with both diseases simultaneously (supplementary data). 82% of the S2B candidates identified with APID3 alone are also found with the merged network APID3HuRI (Fig S3E). Though APID3HuRI candidates captured only 46% of the ones obtained with HuRI alone, the S2B candidates only identified in HuRI or APID3 separately have lower median S2B scores than those found simultaneously in APID3HuRI. Therefore, the use of a combined network returns the most robust candidates of the individual network analysis. Additionally, APID3HuRI also identifies new S2B candidates (Fig S3E), showing that the combination of both networks produced different shortest paths that uncovered possibly relevant proteins.

S2B candidates are associated with related diseases

According to our hypotheis, S2B candidates may be causal, modifiers or directly involved in the phenotypes common to both diseases. It is then logical to expect that some of these candidates may also be associated with other diseases that share phenotypic fetures or affected pathways. According to the DisGenet database, 146 out of the 232 S2B MND candidates are also associated with at least one disease or pathological phenotype (gene-disease associations supported only by text mining were discarded). This set of candidates is actually statistically enriched in associations with 61 diseases (Hypergeometric test, FDR<0.05, complete list in Supplementary data). The disease enrichment is dominated by 27 cancer related conditions. This may be explained by an intrinsic bias in gene-disease association databases, but also by our previous observation that candidate proteins are enriched in cancer related processes like DNA repair and cell cycle. More interestingly, S2B candidates are enriched in 6 neurological, 2 mental and 3 muscular disorders. This prompted us to analyse the interactions of candidate genes and

these three types of disease by building a bipartite network with two distinct types of nodes (genes and diseases), where edges only connect nodes of different types (Fig S4).

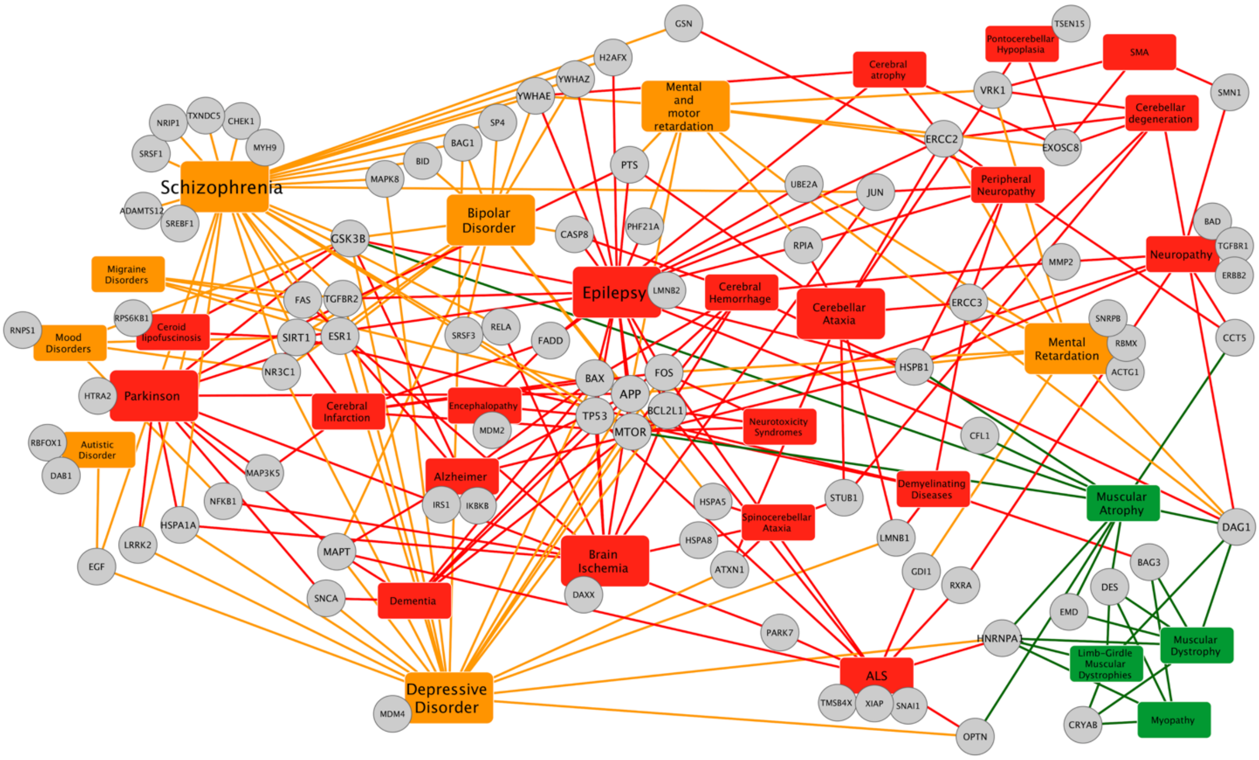

Fig S4. S2B candidate-disease interactions. Diseases associated with S2B candidate proteins (gray nodes) were retrieved from the DisGenet database. Only disease associations with a score higher than 0.08 were used, which discards association based solely on text-mining evidence. All retrieved disease associations are available in Supplementary data. Only associations with Mental (orange nodes), Neurological (red nodes) or Muscular (green nodes) related diseases are represented. Some disease denominations were manually edited to merge subtypes of the same disease. Only diseases associated with at least 3 S2B candidates were included in the network.

Out of a total of 232 S2B candidates, 93 have at least one association with neurological, mental or muscular diseases. In 1000 random sets of 232 genes from the interactome (including 5 SMA seeds, 19 ALS seeds and 2 genes associated with both ALS and SMA, mimicking the composition of the S2B candidate set) the number of genes associated with these types of disease was always significantly lower lower than this (median of 57, 95% confidence interval: [45, 69]). This indicates that S2B candidates are significantly enriched ($p<0.001$) in genes associated with neurological, mental and muscular diseases.

Mental disorders present the highest number of S2B gene-associations (Schizofrenia (28), Depressive disorders (21) and Bipolar disorder (14)). A large diversity of neurological diseases is represented in the network, including neurovascular related (Brain Ischemia (11), Cerebral Hemorrage (7), Cerebral Infarction (6)) and neurodegenerative diseases

(Parkinson(11), Alzheimer (8), Cerebellar Ataxia (8), Demyelinating diseases (5), Spinocerebellar Ataxia (4), Ceroid Lipofuscinosis (3) and Pontocerebellar Hypoplasia (3), besides ALS (12) and SMA (3)). Muscular related disorders are represented by Muscular Atrophy (10), Muscular Dystrophy (6), Limb-girdle Muscular Dystrophy (4) and Myopathy (4). Gene-wise, APP (11), GSK3B (9), MTOR (9), BAX (7), DAG1 (7), ERCC2 (7) and SIRT1 (7) have the higher number of disease associations. More interestingly, BAG3, CCT5, DAG1, GSK3B, HNRNPA1, HSP1B, MTOR and OPTN are simultaneously associated with neurological and muscular diseases.

The association of a high number of S2B candidates with other diseases related with ALS and SMA is an independent observation that supports the ability of the S2B method to identify functionally relevant genes in disease module overlaps.

Correlation of *S2B* with node degree and betweenness

Figure S5 explores the relation between S2B values and degree or betweenness centralities, both in the complete interactome (Figure S5 A and C) or in the S2B candidate subnetwork (Figure S5 B and D).

Figure S5 A and C show that the proteins with highest S2B value are not the ones with highest degree or betweenness. More significantly, among the proteins that pass the specificity score filtering, the proteins with higher S2B value also have higher degree and betweenness, but among the proteins that do not pass the specificity filtering, there are proteins with much higher degree and higher betweenness. In our S2B candidate subnetwork (Figure 3) all proteins have both specificity scores higher than 0.90. Still, some highly connected proteins pass this filter. These highly connected proteins may generally have high S2B values in randomized networks, but the value obtained with the ALS and SMA seeds is still higher than 90% of the randomizations.

Figure S5C shows the relation between S2B values and general betweenness. Although there is a positive correlation between the two measures, the nodes with highest betweenness are not the ones with highest S2B values. Taking into account the specificity score values, many high betweenness nodes are further discarded.

When node degree and betweenness are recomputed for the S2B candidate subnetwork, nodes with highest S2B are also the nodes with higher degree (Figure S5B) and higher betweenness (Figure S5D) of the complete subnetwork. Conversely, among the candidate

nodes with lower S2B values, there is a significant dispersion of degree and betweenness values.

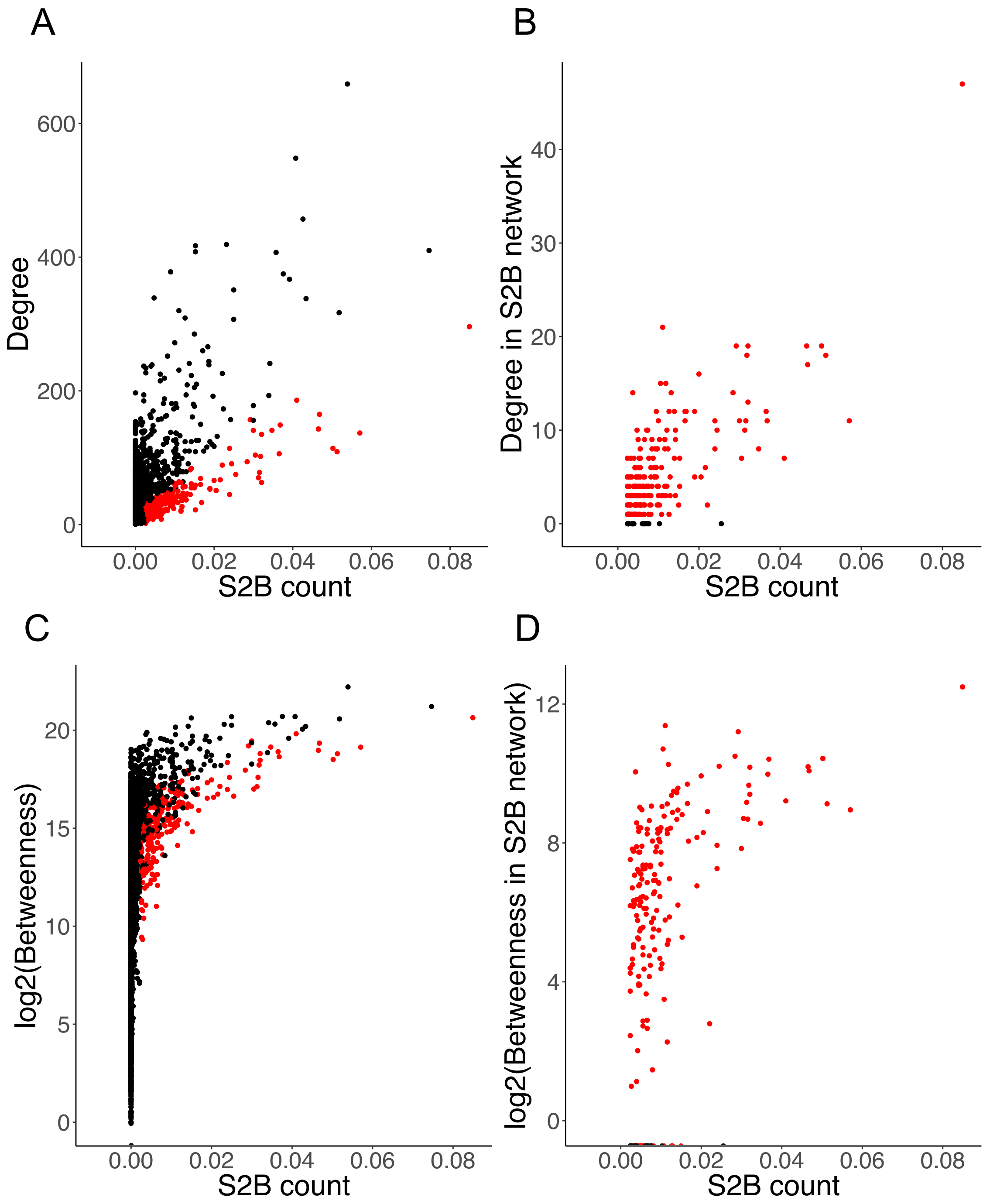

Fig S5. Correlation of *S2B* with node degree and betweenness. (A) Plot of node degree in the complete interactome versus *S2B*. **(B)** Plot of node degree in the candidate network versus *S2B*. **(C)** Plot of node betweenness in the complete interactome versus *S2B*. **(D)** Plot of node betweenness in the candidate network versus S2B. Red dots represent proteins with both specificity scores higher or equal to 0.90.

S2B performance with artificial disease modules

To complement the results presented in Figure 1 of the main text, supplementary figure S6 presents the average number of neighbors in the overlap as a function of S2B rank and the effect of seed number, random edges or seeds in the Recall of S2B predictions.

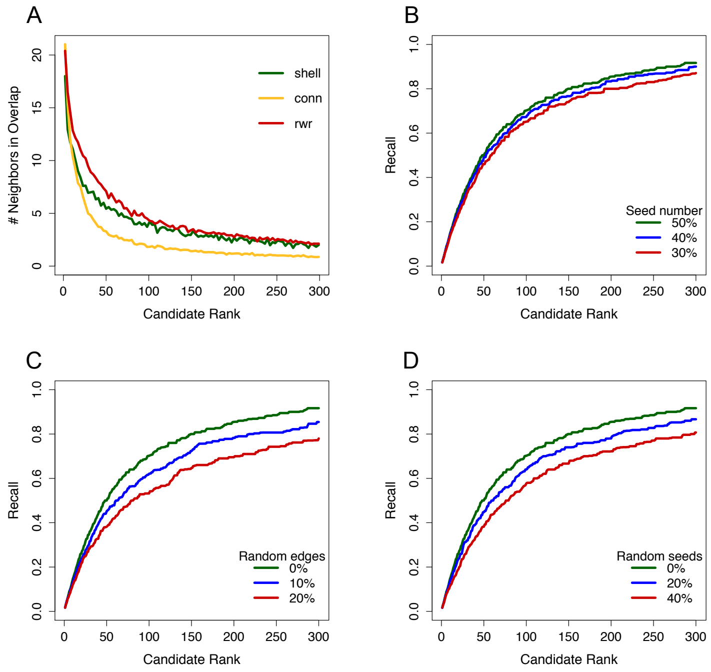

Fig S6. *S2B* performance with artificial modules. (A) Average number of direct neighbors in the overlap protein sets as a function of *S2B* decreasing rank. Three models of disease modules were tested: shell, connectivity (conn) and random walk with restart (rwr) based modules. **(B)** *S2B* recall upon reduction of the fraction of module proteins used as seeds. **(C)** *S2B* recall upon randomly rewiring a fraction of network edges. **(F)** *S2B* recall upon replacing a fraction of input seeds by random proteins. In all plots, values are averages of S2B candidates in three consecutive ranks. Recall is the fraction of proteins in the overlap between the two modules that have an *S2B* rank lower or equal to the candidate rank ploted. In A, 95 pairs of shell modules, 355 pairs of conn modules and 200 pairs of rwr modules were evaluated. In B, C and D, 50 pairs of shell modules were used. Shell modules have between 200 and 400 nodes, while conn and rwr modules have 250 nodes. The overlap between two modules is always between 50 and 125 nodes. In A, C and D, a 50% random sample of each module was used as seeds.

References

1. Hamosh, A. *et al.* Online Mendelian Inheritance in Man (OMIM): a knowledgebase of human genes and genetic disorders. *Nucleic Acids Res.* **30,** 52–55 (2002).

2. Piñero, J. *et al.* DisGeNET: a discovery platform for the dynamical exploration of human diseases and their genes. *Database* **2015,** (2015).

3. Rual, J. F. *et al.* Towards a proteome-scale map of the human protein-protein interaction network. *Nature* **437,** 1173–1178 (2005).

4. Venkatesan, K. *et al.* An empirical framework for binary interactome mapping. *Nat. Methods* **6,** 83–90 (2009).

5. Yu, H. *et al.* Next-generation sequencing to generate interactome datasets. *Nat. Methods* **8,** 478–480 (2011).

6. Rolland, T. *et al.* Resource a proteome-scale map of the human interactome network. *Cell* **159,** 1212–1226 (2014).

7. Yang, X. *et al.* Widespread Expansion of Protein Interaction Capabilities by Alternative Splicing. *Cell* **164,** 805–17 (2016).

8. Alonso-Lopez, D. *et al.* APID interactomes : providing proteome-based interactomes with controlled quality for multiple species and derived networks. *Nucleic Acids Res.* **44,** 529–535 (2016).

9. Csárdi, G. & Nepusz, T. The igraph software package for complex network research. *InterJournal* **Complex Sy,** 1695 (2006).

10. Ghiassian, S. D. *et al.* A DIseAse MOdule Detection (DIAMOnD) Algorithm Derived from a Systematic Analysis of Connectivity Patterns of Disease Proteins in the Human Interactome. *PLOS Comput. Biol.* **11,** e1004120 (2015).

11. Köhler, S., Bauer, S., Horn, D. & Robinson, P. N. Walking the Interactome for prioritization of candidate disease genes. *Am. J. Hum. Genet.* **82,** 949–958 (2008).

12. Yu, G., Wang, L.-G., Han, Y. & He, Q.-Y. clusterProfiler: an R Package for Comparing Biological Themes Among Gene Clusters. *Omi. A J. Integr. Biol.* **16,** 284–287 (2012).

13. Brito, G. C. & Andrews, D. W. Removing bias against membrane proteins in interaction networks. *BMC Syst. Biol.* **5,** (2011).
